# Factors affecting long acting and permanent contraceptive methods utilization among HIV positive married women attending care at ART clinics in Northwest Ethiopia

**DOI:** 10.1186/s13690-018-0294-0

**Published:** 2018-07-16

**Authors:** Abebaw Addis Gelagay, Digsu Negese Koye, Hedija Yenus Yeshita

**Affiliations:** 1 Department of Reproductive Health, College of Medicine and Health Sciences, University of Gondar, Gondar, Ethiopia; 2 Department of Epidemiology and Biostatistics, College of Medicine and Health Sciences, University of Gondar, Gondar, Ethiopia

**Keywords:** Long acting and permanent contraceptive methods, Unintended pregnancy, Human Immuno-deficiency virus, Married women, Ethiopia

## Abstract

**Background:**

Globally, unintended pregnancy has been very high accounting for 27% of maternal deaths. Different studies noted that nearly half of HIV positive women who gave unintended birth were using contraceptive methods prior to their unintended pregnancy. This implies that contraceptive failure contributes to unintended pregnancy. Long-term and permanent contraceptive methods are safe and effective contraceptive options. In women who are using long acting and permanent methods, the unintended pregnancy rate is very low and it is almost the same both in typical and perfect users. However, there is limited information on factors that affect long acting and permanent contraceptive methods utilization among Human Immuno-deficiency Virus (HIV) positive women in Ethiopia. Therefore, the purpose of this study was to assess long acting and permanent contraceptive utilization and its associated factors.

**Methods:**

An institution-based cross-sectional study was conducted among 505 married women attending care at Anti Retroviral Therapy (ART) clinics in Bahir Dar from March 16, 2014 to April 15, 2014. The data were collected using a structured and interviewer administered questionnaire. Both bivariate and multivariate logistic regressions were used to identify associated factors.

**Results:**

A total of 505 married women participated in the study with a response rate of 99.6%. The utilization of long acting and permanent contraceptive methods (LAPMs) was 27.5% [95% CI, 23.8–31.5]. The multivariate analyses showed that women who were getting pre- anti retroviral therapy (Pre-ART) services [Adjusted Odds Ratio = 2.65, 95% confidence interval: 1.44, 4.86], had spousal discussion on family planning sometimes [Adjusted Odds Ratio = 6.03, 95% CI:2.42–15.01] and frequently [Adjusted Odds Ratio = 6.61, 95% confidence interval: 2.49–17.47], had previous experience on long acting contraceptive methods [Adjusted Odds Ratio = 9.06, 95% confidence interval: 5.38–15.26], no exposure to myths about LAPMs [Adjusted Odds Ratio = 2.07, 95% confidence interval: 1.24–3.45], had birth intention after 2 years [Adjusted Odds Ratio = 6.95, 95% confidence interval: 3.35–14.42], and no such intention [Adjusted Odds Ratio = 7.60, 95% confidence interval: 3.77–15.34] were factors significantly associated with utilization of long acting and permanent contraceptive methods.

**Conclusion:**

The utilization of long acting and permanent contraceptive methods was relatively high. Discussion with partners on family planning, previous experiences of LAPMs, not hearing myths about LAPMs, women not started ART, and no birth intention were positively associated with LAPMs utilization. It is therefore recommended that health service providers need to make couples counseling on FP, undergo behavioral change communication (BCC) to avoid misconceptions/myths regarding LAPMs. Further research is also recommended to address the gaps mentioned in the limitation section and to explore the reason/s for not using LAPMs (qualitative study).

**Electronic supplementary material:**

The online version of this article (10.1186/s13690-018-0294-0) contains supplementary material, which is available to authorized users.

## Background

HIV/AIDS has continued to be a public health problem. UNADS report showed that in 2013, 35 million people were estimated to live with HIV worldwide. Out of these, approximately 3.2 million were children under 15 years of age, and 91% of these children were living in Sub-Saharan Africa [1]. Although there has been reductions in new HIV infections among children, there were still a significant number of new cases. Out of the globally estimated 240,000 newly HIV infected children, 210,000 were living in Sub-Saharan Africa, where women account for 58% of the total number of people living with the virus [1, 2].

Preventing unintended pregnancy among women living with HIV is essential to decrease pregnancy-related morbidity and mortality as well as mother to child HIV transmission. Family planning is one of the four pillars of guidance on the prevention of the mother to child transmission (MTCT) of the virus. Meeting the family planning goals of women living with HIV through appropriate counseling and contraceptives services will optimize health outcomes for women and reduce the potential for HIV transmission to their children [3].

Unintended pregnancy remains an alarming global public health problem with its subsequent socioeconomic impact on individuals, families, and the society [4]. Though there is a considerable variation in the prevalence of unintended pregnancy across regions, the global burden is very high (40% in 2012) [5] and responsible for 27% of maternal deaths [6]. Different cross-sectional studies around the globe noted that there has been a high prevalence of unintended pregnancy for example, 69.2% in Swaziland [7], 56% in Canada [6], 62.7% in Rwanda [8], 59% in Kenya [9], and 61.6% in South Africa [10] among HIV positive women highlighting the need for effective contraceptive utilization [11]. Unintended pregnancy is a common problem in Ethiopia. The 2011 Ethiopian Demographic and Health Survey (EDHS) report showed that there was 28.3% of unintended pregnancy) [12]. A study conducted in Kersa, Ethiopia, noted that 27.9% of pregnancies were unintended [13]. A study done in West Wolega, Ethiopia, also noted that 36.5% of the pregnancy was unintended [14].

Different studies at different parts of the globe noted that a large proportion of unintended pregnancy is due to poor use of short-term hormonal methods. For example, a study conducted in Swaziland documented that half of the HIV positive women reported that they were using family planning methods when they became pregnant. A study done in sub-Saharan Africa identified that a significant proportion of unintended pregnancies was due to poor use of short acting hormonal methods [15]. As revealed in a study done in Zimbabwe, nearly half (47.8%) of women who had unintended births were using contraceptives prior to their pregnancies [11]. A study done here in Ethiopia showed that contraceptive method-failure shared 31.5% of the unintended pregnancy [14]. Generally, both contraceptive failure and unmet needs for family planning contribute to unintended pregnancy. This highlights the need for increased investment in safe and effective contraceptive methods.

Long acting and permanent family planning methods (LAPMs) are modern contraceptive methods that prevent pregnancy for more than 1 year and include long acting reversible contraceptive methods (Intrauterine device and sub dermal implants) and permanent contraceptive methods (Tubaligation and Vasectomy), also called sterilizations. The World Health Organization categorized contraceptive methods on the basis of their efficacy. LAPMs (IUCDs, Implants, Vasectomy, and Tubaligation) have the highest efficacy; combined hormonal and progesterone only methods have moderate efficacy, while barrier methods and fertility awareness are the least effective [16].

Since LAPMs do not require adherence and avoid the adverse effects and health risks of estrogen containing contraceptives, they are safe and effective contraceptive options. In women who are using long acting and permanent methods, the unintended pregnancy rate is very low and it is almost the same both in the typical users, when the method is not always used consistently and according to instructions, (0.05 to 3.0 cases per 100 women), and perfect users, when the method of family planning is used consistently and according to instructions, (0.05 to 0.6 cases per 100 women). So, long acting contraceptive methods are appropriate for a wide range of women seeking to limit or space birth. Despite their safety and efficacy, these methods are underutilized (less than 5%) in Ethiopia in the general community irrespective of HIV status [12]. Information on factors affecting LAPMs utilization among women living with HIV is limited in the study area and in Ethiopia in general. Therefore, the purpose of this study was to assess factors that affect LAPMs use.

## Methods

An institution-based cross-sectional study was conducted among 15–49 years old married HIV positive women attending services at ART clinics of public health institutions in Bahir Dar.

### Study area and period

The study was conducted in Bahir Dar, northwest Ethiopia, from March 16th to April 15th, 2014. Bahir Dar, the capital of the Amhara Region, is located at 565 km to the northwest of Addis Ababa, the capital of Ethiopia. Divided into nine sub-cities, Bahir Dar had 288,200 inhabitants of whom 147,597 were females, according to BOFED 2014. In the city, there were one public and one private general hospitals in addition to ten health centers. Only three of which had active ART clinics. So, the respondents received services from Feleg-Hiwot Referral Hospital (public), Bahir Dar, Han, and Abay health centers. All of health the institutions were providing family planning services. All long acting reversible contraceptives methods (Implants and Intrauterine device) were available in the health centers while both long acting reversible contraceptives methods and permanent methods were accessible in the hospital.

### Study population

The single population proportion formula was used to calculate the sample. The utilization of LAPMs among the general population of the study conducted in Goba town, Southeast Ethiopia (8.7%) was used to calculate the sample size. By considering standard normal distribution, Z-value at 95% confidence level (CI) is 1.96. Three percent (3%) was considered as margin of error. After considering 10% allowance for non response, the final sample size planned was 507. Sample size was also calculated for factors. However, all were less than the sample estimated for the dependent variable. The total number of reproductive age (15–49 years) married women who were served at each service delivery point during the 1 month period was estimated based on a 1 week preliminary survey. The sample size was proportionally allocated to each service delivery point. The study population were selected using systematic random sampling technique. Ahead of the beginning of data collection, the first sample was chosen by lottery method and then the other study participants were selected with an interval of seven. Since, the sample was representative to all married HIV positive women attending care at ART clinics in Northwest Ethiopia, the findings were generalizable to these people. Reproductive age women who were seriously ill (unable to give the required information) were excluded.

### Variables of the study

Outcome variable: Long acting and permanent contraceptive methods (LAPMs) utilization.

Exposure variables: The exposure variables included demographic variables (age, residence, number of a live children, birth intention), socio-economic variables (religion, occupation, women education, partner education, monthly family income, ownership of TV/Radio), and clinical and method related factors (ART status, Past experience for LAPMs, myths heard about LAPMs, spousal discussion on Family planning, advertisement ever heard).

- **Long acting and permanent contraceptive methods (LAPMs) utilization**: Utilization of any of the long acting and permanent contraceptive methods during the data collection period by dichotomizing their response as Yes or No.
- **Currently married women**: Women whose marital status were married at the time of data collection.
- **Myths heard**: When women had ever heard any rumor or misconceptions about LAPMs, for example. Myths related to Intra-uterine device: it can cause cancer, the device will be embedded in fetal brain if pregnancy happens; myths related implants: it causes sterility, Arthritis, etc.
- **Typical users**: are family planning users who do not always used the method/s consistently and according to instructions.
- **Perfect users**: are family planning users who used the method/s consistently and according to instructions.
- **Past experience for LAPMs**: It is when a women had ever used LAPMs before current used method.
- **Ownership of TV/Radio**: When a women or a couple have ever had Television and/or Radio.
- ART status: The ART status of the respondents was explained by the initiation of anti-retro viral therapy (ART) and the response was either “on ART” or “not on ART”.

### Data collection tool and process

A structured questionnaire was first prepared in English and then translated to the local language, Amharic, and then re-translated to English language by language experts. Five nurses were employed to collect data, and one public health officer participated as a supervisor of the data collection. Data collectors and the supervisor were given training on data collection tool and the procedure by the principal investigator. The tool was pre-tested on 30 individuals outside the study area, and few amendment were made after the pre-test. A systematic random sampling technique with an interval of seven in their order of arrival for services was used. The required data were collected after obtaining verbal consent. The supervisor and the principal investigators supervised the data collection process and checked the data completeness on daily bases.

### Data processing and analysis

The data were checked and coded manually, and entered into EPI Info version 3.5.3 and exported to SPSS version 20 for analysis. Each explanatory variable was first analyzed by using bivariate logistic regression. All the independent variables (age, residence, occupation, religion, educational status of women, educational status of women, family monthly income, LAPMs advertisement heard, ART status, spousal discussion on FP, main decider on contraceptive use, previous use of LACMs, myths heard about LAPMs, number of alive children, and birth intention) that we considered for bivariate analysis were also considered for the multivariable analysis to see their independent association with LAPMs utilization.

Multicollinearity and interaction were assessed for covariates. Multicollinearity was found between ownership of radio &/or radio and advertisement heard about LAPM and between total live births and number of alive children (see Additional file 1). Hence, ‘ownership of radio &/or television’ and ‘total live births’ were removed from the final model (multivariable analysis). Interaction was observed between ‘discussion with husband on FP’ and ‘main decider on contraceptive use’ and between ‘age’ and ‘birth intention’ (see Additional file 2). However, none of them retained in the final model (see Additional file 3). Backward stepwise logistic regression method was used. The goodness of fit for the final model was 0.829.. In the multivariable analysis, adjusted odds ratio (AOR) with 95% CI was used to assess statistical significance and strength.

## Results

A total of 505 women participated in this study with a response rate of 99.6%. The mean age of the participants was 31.59 years (SD = 5.44). The majority (84.4%) of the participants were Orthodox Christians. The median monthly family income was 62.00USD (Inter Quartile Range = 99USD). Four hundred nineteen (83%) of the participants had television sets and/or radio receivers (Table 1).

**Table 1:** Socio-demographic and economic characteristics of reproductive age women attending ART care in public health facilities at Bahir Dar City, Northwest Ethiopia, 2014

Variables	Category	Frequency	Percentage
Age (in years)	15–24	42	8.3%
	25–34	298	59.0%
	35 and above	165	32.7%
Religion	Orthodox	426	84.4%
	Muslim	55	10.9%
	Protestant	22	4.4%
	Catholic	2	0.4%
Current place of residence	Urban	456	90.3%
	Rural	49	9.7%
Occupation	House wife	186	36.8%
	Daily laborer	101	20.0%
	Government employee	98	19.4%
	Merchant	101	20.0%
	Others^a^	19	3.8%
Educational status of women	No formal education	221	43.8%
	Primary school (grade 1–8)	111	22.0%
	Secondary school (grade 9–10)	81	16.0%
	Preparatory, College and above	92	18.2%
Educational status of the husband	No formal education	131	25.9%
	Primary school (grade 1–8)	122	24.2%
	High school (grade 9–10)	113	22.4%
	Preparatory, College and above	139	27.5%
Family monthly income (USD)^b^	≤30	132	26.1%
	31–62	115	22.8%
	63–129	148	29.3%
	≥130	110	21.8%
Ownership of TV & Radio	Yes	86	17.0%
	No	419	83.0%
Number of children ever born	0–2	376	74.5%
	3 and above	129	25.5%
Number of alive children	0–1	242	47.9%
	2–3	233	46.1%
	4 and above	30	5.9%

^a^ Private employee, Farmer, and students. 1USD = 19.37 ETB

^b^ Income was classified according to the quartile classification

A significant number, 382 (75.6%), of the study participants were using modern contraceptive methods. Long acting and permanent contraceptive methods utilization was 27.5% [95% CI: 23.8–31.5] (16.4% for limiting while 11.1% for spacing). Among long acting and permanent contraceptive method users, 116 (83.5%), 22(15.8%), and 1(0.7%) were using implants, IUD, and tubaligation respectively.

Most, 496 (98.2%), of the participants had actually heard about long acting and permanent contraceptive methods. The most known LAPMs were Implants (96%) and Intra uterine contraceptive method (86.7%). However, Tubaligation and Vasectomy were so less popular among the respondents that only 85 (16.8%) and 36 (7.1%) of the participants mentioned them. The mass media (85%) and health professionals (83%) were the main sources of information about the LAPMs. Out of the participants, 151 (29.9%) had past experience of LAPMs utilization, while 281 (55.6%) heard myths about LAPMs.

### Factors associated with utilization of LAPMs

The multivariate analysis showed that ART status, FP discussion with husband, previous experience of LAPMs, myths heard about LAPMs, and birth intention were significantly associated with the utilization of long acting and permanent contraceptive methods. On the contrary, age, residence, occupation, LAPMs advertisement heard, number of live children they had, and main decider on contraceptive utilization were not significantly associated with utilization of LAPMs.

The women who were getting pre-ART services were two point six times more likely [AOR = 2.65, 95% CI: 1.44, 4.86] to utilize LAPMs than women on ART. Women who had sometimes discussed on family planning issues with their husbands were six times more likely [AOR = 6.03, 95% CI:2.42–15.00] to utilize LAPMs than those who didn’t discuss with their husbands. Similarly, women who had frequently discussed on family planning issues were six point six times more likely [AOR = 6.61, 95% CI: 2.49–17.47] to utilize LAPMs than those who didn’t discuss with their husbands. Women who had previous experience of long acting reversible contraceptives were nine times more likely [AOR = 9.06, 95% CI: 5.38–15.26] to utilize LAPMs than women who had no past experience. Women who hadn’t heard myths about LAPMs were two times more likely [AOR = 2.07, 95% CI: 1.24–3.45] to utilize LAPMs compared with women who had heard myths. Women who had birth intention after 2 years were almost seven times more likely [AOR = 6.95, 95% CI: 3.35–14.42] and women who had no birth intention were seven point six times more likely [AOR = 7.60, 95% CI: 3.77–15.34] to utilize LAPMs compared to women who had intention to give birth within 2 years (Table 2).

**Table 2:** Bivariate and multivariable analyses of factors associated with utilization of LAPMs among women attending ART care in public health facilities at Bahir Dar City, Northwest Ethiopia, 2014

Independent variables		LAPMs utilization		COR (95% CI)	AOR (95% CI)
		Yes	No		
ART status	Pre-ART	36	60	1.78 (1.11–2.85)	2.65(1.44–4.86)
	ART	103	306	1.00	1.00
Spousal discussion on FP	No	14	84	1.00	1.00
	Some times	80	203	2.37(1.27–4.41)	6.03(2.42–15.00)
	Frequently	45	79	3.42(1.74–6.71)	6.61(2.49–17.47)
Previous Use of LACMs	Yes	80	71	5.63(3.69–8.61)	9.06(5.38–15.26)
	No	59	295	1.00	1.00
Myths heard about LAPMs	Yes	61	220	1.00	1.00
	No	78	146	1.93(1.29–2.86)	2.07(1.24–3.45)
Birth intention	Want within two year	14	155	1.00	1.00
	Want after two year	43	71	6.71(3.45–13.04)	6.95(3.35–14.42)
	No more children wanted	82	140	6.49(3.52–11.95)	7.60(3.77–15.34)

Note: 1.00 = Reference; Hosmer Lemeshow test = 0.829

## Discussion

This study revealed that more than a quarter (27.5%) of HIV positive reproductive age married women utilized LAPMs. ART status, spousal discussion on family planning, previous use of long acting contraceptives, myths heard about LAMPS, and women’s birth intention were the factors associated with LAPMs utilization.

LAPMs utilization in this study was higher than the studies conducted in Debre Markos, northwest Ethiopia (19.5%) [17], Gimbie, west Ethiopia (15.6%) [18], Mekelle, north Ethiopia (12.3%) [19], Debre Tabor, northwest Ethiopia (9.2%) [20], Goba, southeast Ethiopia (8.7%) [21], Jinka, southern Ethiopia (7.3%) [22], Batu, central Ethiopia (3%) [23], and western Ethiopia (20%) [24].

Possible reasons for these variations could be difference among the study participants in that the respondents of this study had ART follow up and frequent exposure to health education programs and family planning counseling. In this study, 98.2% of the respondents heard about LAPMs, while, most of the other studies were community based and this could cause differences in awareness on the multifaceted advantages of long acting and permanent contraceptive methods over short acting ones.

Specifically, the difference with the study done in Gimbie town might be due to two main reasons. The first reason could be the study done in Gimbie considered all reproductive age women irrespective of their sexual status/exposure that including sexually inactive women could reduce the prevalence of contraceptive utilization in general and LAPMs utilization in particular. This reason could also be a reason for a study conducted in Jinka town. The second reason could be- the study participants were those who were on ART that didn’t include those who were using Pre-ART services but not started ART on the moment. In this study, it was identified that the prevalence of LAPMs varied by ART status (higher prevalence of LAPMs was observed among pre-ART service users than those who were on ART). Another possible reasons for the difference with the study in Jinka could be time difference and difference in the study population that they did the study among all reproductive age women irrespective of HIV serostatus. However, the birth intention among HIV positive and HIV negative women is not the same. It is commonly seen higher among HIV negative than HIV positive ones. This could affect the prevalence of LAPMs.

The utilization of LAPMs in this study was in line with the study done in Mahabad, Iran (27.7%) [25]. However, this finding was inconsistent with the findings of studies conducted in different areas. The utilization in this study was lower than those of studies done in Kampala district, Uganda (31.7%) [26] and results from nationally representative surveys in southern Africa (South Africa and Zimbabwe) (34%) [27]. This variation might be due to differences in the acceptance of LAPMs, which is, in turn, affected by the quality of family planning counseling services and community’s perception towards LAPMs. Moreover, the latter might be influenced by the exposure to myths. Indeed, in this study, more than half (55.6%) of the study participants have heard myths about LAPMs. Generally, socio-demographic characteristics might be the cause for the observed difference. In this study, respondents’ ART status was found to have statistically significant association with the utilization of LAPMs. Respondents who were getting pre-ART services were 2.6 times more likely to utilize LAPMs compared to those who were on ART services. This might be due to differences in clinical condition. HIV positive women who were not eligible for ART were usually found in a relatively good clinical condition. Thus, their sexual interest might be intact. Another alternative reason could be due to difference in birth intention that women who were in ART might have birth intention than those who didn’t start ART. In this study, the disaggregated result on ART status noted that higher proportion of women who didn’t start ART (61.7%) had no more birth intention at all than women who were on ART (41.4%). This idea is also supported by different studies [28–30].

One form of male involvement in family planning is providing support and making open discussions with partners. Studies done in Malaysia and Ghana documented that women who had discussion with their spouse about family planning had better use of the methods [31, 32]. Another study done in Shashemene, Ethiopia also noted that lack of discussion between partners negatively influences LAPMs use [33]. In this study, spousal discussion on modern family planning methods was significantly associated with utilization of LAPMs. Respondents who sometimes discussed with their partners were six times more likely, and those who frequently discussed were 6.6 times more likely to use long acting and permanent contraceptive methods than those who had no spousal discussions. This association was also seen in studies conducted in Debre Tabor [20], Goba [21] and Butajira district, Ethiopia [34].

In this study, it was noted that previous use of long acting contraceptive methods was strongly associated with the utilization of LAPMs. Respondents who had used long acting contraceptive methods previously were about nine times more likely to utilize LAPMs compared to those who hadn’t used. This association was also seen in studies conducted in Goba town, southeast Ethiopia [21] and Uganda [26]. This could be due to the following reasons: Respondents who had used LAPMs might have frequent and detailed discussions with service provider/s specifically on the methods they were using; those who had used LAPMs might find the methods convenient and appreciate the advantages of the methods, and might not be influenced by misperceptions on LAPMs.

In this study, it is identified that myths had association with utilization of LAPMs. Respondents who had never heard myths about LAPMs were two times more likely to utilize LAPMs than those who had heard myths. The reason might be because of the fact that individuals, more importantly new users, who have heard myths could have misperceptions which make them have negative attitude and poor interest in using the service. Studies conducted in urban Africa [35] and Kenya [36] noted that myths about family planning methods are barriers to use the methods. Since such misperceptions were prevalent (55.64%) in this study and are not limited in this study area that requires behavioral change interventions.

Child bearing intention was found to be a strong predictor of utilization of LAPMs. In this study, participants who had child bearing intention after 2 year were almost seven times more likely and those who had no desire for more children were seven point six times more likely to utilize LAPMs compared to those who had child bearing intention within 2 years. The same association was seen in studies conducted in Debre Markos [17], Mekelle [19], Debre Tabor [20], Ethiopia, and Mahabad, Iran [25]. The possible reason for this could be that when people are satisfied with the desired number of children or have desire after long time period, they might not want to have frequent visits for contraceptive service and might choose LAPMs. Additionally, though method choice is the right of service users, usually service providers might recommend them to take LAPMs accordingly.

The implication of the findings of this study is that the prevalence of LAPMs can be further increased by creating platform for couples to discuss on family planning through male partners involvement in family planning counseling. Additionally, undergoing behavioral change communication (BCC) to divert the false beliefs regarding LAPMs can contribute for a high prevalence of LAPMs. Though the service (LAPMs) should be available for all women, it can be more effective if emphasis is given for women who have no birth intention or have intention after long time. More effort is required during FP counseling to get new LAPMs adopters.

Because of the sensitive nature of enquiring whether they were sexually active or not, and using health professionals who were working in the same ART clinics as data collectors could make unmarried women not truly disclose whether they were sexually active or not. Hence, this study focused only on married reproductive age women. This is an observer bias that might lower the prevalence of the LAPMs in this study. This is because birth intention outside marriage is not common in Ethiopia that sexually active unmarried women might choose LAPMs. Thus, the prevalence of LAPMs could not be inferred to all sexually active HIV positive women. As another possible limitation, it is worth mentioning that some important variables have not been considered or have been missed along the study. For example, the authors did not consider respondents’ HIV/AIDS stage and clinical conditions including CD4 count. Hence, the authors couldn’t stratify the analysis by client’s clinical status which limit the possibility of identifying additional predictors for LAPMs utilization. The authors want to mentioned that formal study on observers agreement was not made but attempts were taken to avoid inter-observers differences in understanding the data collection instrument (questionnaire) by providing training for the data collectors and supervisors, doing pre-test, and through day to day supervision during data collection. This study might slightly be subjected to social desirability bias that might inflate the prevalence. But, adequate information were given to participants about the critical importance of telling the actual information. Finally, since it is a cross section study, the possible limitation might be establishing the cause and effect relation between the dependent and independent variables.

## Conclusion and recommendations

The utilization of long acting and permanent contraceptive methods in this study was relatively high. Discussions with partners on family planning, previous experiences of LAPMs, not hearing myths about LAPMs, women not started ART, and no birth intention were positively associated with LAPMs utilization. It is therefore recommended that health service providers need to make couples counseling on FP that may create a platform and an entry point for couples to discuss on their fertility, contraceptive use, and method choice. Additionally, service providers should undergo behavioral change communication (BCC) to avoid misconceptions/myths regarding LAPMs. Further research is also recommended to address the gaps mentioned in the limitation section and to explore the reason/s for not using LAPMs (qualitative study).

## Additional files

Additional file 1: A table that shows multicollinearity analysis. (DOCX 27 kb)

Additional file 2: Interaction tables. (DOCX 61 kb)

Additional file 3: Hosmer and Lemeshow Test and the multivariable analysis output. (DOCX 27 kb)

## Abbreviations

AIDS: Acquired Immuno-deficiency Diseases Syndrome
AOR: Adjusted odds ratio
ART: Antiretroviral treatment
FP: Family planning
HIV: Human Immunodeficiency virus
IUCDs: Intrauterine contraceptive devices
LAPMs: Long acting and permanent contraceptive methods
