# Supplementary material for: Factors affecting long acting and permanent contraceptive methods utilization among HIV positive married women attending care at ART clinics in Northwest Ethiopia
Source: Arch Public Health. 2018 Jul 16;76:47. doi: 10.1186/s13690-018-0294-0 (PMC6047118; doi:10.1186/s13690-018-0294-0)
Supplement: Supplementary file 1 — A table that shows multicollinearity analysis. (DOCX 27 kb) [file 13690_2018_294_MOESM1_ESM.docx]

**Multicollinearity analysis output**

| **Correlations** | | | | | | | | | | | | | | |
| --- | --- | --- | --- | --- | --- | --- | --- | --- | --- | --- | --- | --- | --- | --- |
|  | | AGE | Residence | Religion | Occupation | Income | Ownership of TV & R | LACM advertisement | Have heard myths | FP discussion | Main decider on F/P | Birth intention | TotalLive Births | Number of Alive child. |
| AGE | Pearson Correlation | 1 | .077 | -.068 | -.022 | -.021 | -.034 | -.037 | .005 | -.006 | -.030 | .214^**^ | .332^**^ | .331^**^ |
|  | Sig. (2-tailed) |  | .082 | .127 | .615 | .635 | .445 | .411 | .909 | .895 | .496 | .000 | .000 | .000 |
|  | N | 505 | 505 | 505 | 505 | 505 | 505 | 505 | 505 | 505 | 505 | 505 | 505 | 505 |
| Residence | Pearson Correlation | .077 | 1 | -.040 | -.030 | -.108^*^ | .322^**^ | .330^**^ | -.025 | .194^**^ | -.157^**^ | -.079 | .195^**^ | .194^**^ |
|  | Sig. (2-tailed) | .082 |  | .366 | .496 | .015 | .000 | .000 | .581 | .000 | .000 | .076 | .000 | .000 |
|  | N | 505 | 505 | 505 | 505 | 505 | 505 | 505 | 505 | 505 | 505 | 505 | 505 | 505 |
| Religion | Pearson Correlation | -.068 | -.040 | 1 | .047 | .262^**^ | .138^**^ | .130^**^ | -.006 | -.020 | .038 | -.010 | .039 | .056 |
|  | Sig. (2-tailed) | .127 | .366 |  | .296 | .000 | .002 | .003 | .893 | .654 | .397 | .817 | .385 | .211 |
|  | N | 505 | 505 | 505 | 505 | 505 | 505 | 505 | 505 | 505 | 505 | 505 | 505 | 505 |
| Occupation | Pearson Correlation | -.022 | -.030 | .047 | 1 | .260^**^ | .099^*^ | .126^**^ | .018 | .131^**^ | .043 | -.113^*^ | -.073 | -.079 |
|  | Sig. (2-tailed) | .615 | .496 | .296 |  | .000 | .026 | .005 | .683 | .003 | .332 | .011 | .101 | .076 |
|  | N | 505 | 505 | 505 | 505 | 505 | 505 | 505 | 505 | 505 | 505 | 505 | 505 | 505 |
| Income | Pearson Correlation | -.021 | -.108^*^ | .262^**^ | .260^**^ | 1 | .321^**^ | .307^**^ | .062 | .224^**^ | .180^**^ | -.127^**^ | -.042 | -.003 |
|  | Sig. (2-tailed) | .635 | .015 | .000 | .000 |  | .000 | .000 | .162 | .000 | .000 | .004 | .347 | .938 |
|  | N | 505 | 505 | 505 | 505 | 505 | 505 | 505 | 505 | 505 | 505 | 505 | 505 | 505 |
| Ownership of TV & R | Pearson Correlation | -.034 | .322^**^ | .138^**^ | .099^*^ | .321^**^ | 1 | .776^**^ | .126^**^ | .309^**^ | .266^**^ | -.041 | -.056 | -.041 |
|  | Sig. (2-tailed) | .445 | .000 | .002 | .026 | .000 |  | .000 | .004 | .000 | .000 | .360 | .209 | .359 |
|  | N | 505 | 505 | 505 | 505 | 505 | 505 | 505 | 505 | 505 | 505 | 505 | 505 | 505 |
| LACM advertisement | Pearson Correlation | -.037 | .330^**^ | .130^**^ | .126^**^ | .307^**^ | .776^**^ | 1 | .135^**^ | .227^**^ | .233^**^ | -.002 | -.051 | -.039 |
|  | Sig. (2-tailed) | .411 | .000 | .003 | .005 | .000 | .000 |  | .002 | .000 | .000 | .966 | .252 | .379 |
|  | N | 505 | 505 | 505 | 505 | 505 | 505 | 505 | 505 | 505 | 505 | 505 | 505 | 505 |
| Have heard myths | Pearson Correlation | .005 | -.025 | -.006 | .018 | .062 | .126^**^ | .135^**^ | 1 | .093^*^ | .072 | .013 | .007 | -.044 |
|  | Sig. (2-tailed) | .909 | .581 | .893 | .683 | .162 | .004 | .002 |  | .037 | .106 | .763 | .877 | .325 |
|  | N | 505 | 505 | 505 | 505 | 505 | 505 | 505 | 505 | 505 | 505 | 505 | 505 | 505 |
| FP discussion | Pearson Correlation | -.006 | .194^**^ | -.020 | .131^**^ | .224^**^ | .309^**^ | .227^**^ | .093^*^ | 1 | .522^**^ | -.045 | -.135^**^ | -.110^*^ |
|  | Sig. (2-tailed) | .895 | .000 | .654 | .003 | .000 | .000 | .000 | .037 |  | .000 | .315 | .002 | .014 |
|  | N | 505 | 505 | 505 | 505 | 505 | 505 | 505 | 505 | 505 | 505 | 505 | 505 | 505 |
| Main decider on F/P | Pearson Correlation | -.030 | -157^**^ | .038 | .043 | .180^**^ | .266^**^ | .233^**^ | .072 | .522^**^ | 1 | -.091^*^ | -.027 | -.014 |
|  | Sig. (2-tailed) | .496 | .000 | .397 | .332 | .000 | .000 | .000 | .106 | .000 |  | .040 | .541 | .757 |
|  | N | 505 | 505 | 505 | 505 | 505 | 505 | 505 | 505 | 505 | 505 | 505 | 505 | 505 |
| Birth intention | Pearson Correlation | .214^**^ | -.079 | -.010 | -.113^*^ | .127^**^ | -.041 | -.002 | .013 | -.045 | -.091^*^ | 1 | .405^**^ | .399^**^ |
|  | Sig. (2-tailed) | .000 | .076 | .817 | .011 | .004 | .360 | .966 | .763 | .315 | .040 |  | .000 | .000 |
|  | N | 505 | 505 | 505 | 505 | 505 | 505 | 505 | 505 | 505 | 505 | 505 | 505 | 505 |
| Total Live Births | Pearson Correlation | .332^**^ | .195^**^ | .039 | -.073 | -.042 | -.056 | -.051 | .007 | -.135^**^ | -.027 | .405^**^ | 1 | .867^**^ |
|  | Sig. (2-tailed) | .000 | .000 | .385 | .101 | .347 | .209 | .252 | .877 | .002 | .541 | .000 |  | .000 |
|  | N | 505 | 505 | 505 | 505 | 505 | 505 | 505 | 505 | 505 | 505 | 505 | 505 | 505 |
| Number of Alive child. | Pearson Correlation | .331^**^ | .194^**^ | .056 | -.079 | -.003 | -.041 | -.039 | -.044 | -.110^*^ | -.014 | .399^**^ | .867^**^ | 1 |
|  | Sig. (2-tailed) | .000 | .000 | .211 | .076 | .938 | .359 | .379 | .325 | .014 | .757 | .000 | .000 |  |
|  | N | 505 | 505 | 505 | 505 | 505 | 505 | 505 | 505 | 505 | 505 | 505 | 505 | 505 |
