# Supplementary material for: Factors affecting long acting and permanent contraceptive methods utilization among HIV positive married women attending care at ART clinics in Northwest Ethiopia
Source: Arch Public Health. 2018 Jul 16;76:47. doi: 10.1186/s13690-018-0294-0 (PMC6047118; doi:10.1186/s13690-018-0294-0)
Supplement: Supplementary file 2 — Interaction tables. (DOCX 61 kb) [file 13690_2018_294_MOESM2_ESM.docx]

**Tables that show interaction between independent variables:**

| **Variables in the Equation(Ownership of Radio/Television Vs Advertisement heard)** | | | | | | | | | |
| --- | --- | --- | --- | --- | --- | --- | --- | --- | --- |
|  | | B | S.E. | Wald | df | Sig. | Exp(B) | 95% C.I.for EXP(B) | |
|  |  |  |  |  |  |  |  | Lower | Upper |
| Step 1^a^ | Q109Ownershipofredioortelevision(1) by Q110LACadvertismentheard(1) | .470 | .279 | 2.840 | 1 | .092 | 1.601 | .926 | 2.766 |
|  | Constant | -1.360 | .257 | 27.947 | 1 | .000 | .257 |  |  |
| a. Variable(s) entered on step 1: Q109Ownershipofredioortelevision * Q110LACadvertismentheard . | | | | | | | | | |

| **Variables in the Equation (FP discussion Vs Main decider on contraceptive use)** | | | | | | | | | |
| --- | --- | --- | --- | --- | --- | --- | --- | --- | --- |
|  | | B | S.E. | Wald | df | Sig. | Exp(B) | 95% C.I.for EXP(B) | |
|  |  |  |  |  |  |  |  | Lower | Upper |
| Step 1^a^ | Q201FPdiscussionwithhasband * Q202Maindecideroncontraceptiveuse |  |  | 14.578 | 4 | .006 |  |  |  |
|  | Q201FPdiscussionwithhasband(1) by Q202Maindecideroncontraceptiveuse(1) | .282 | .823 | .118 | 1 | .732 | 1.326 | .264 | 6.649 |
|  | Q201FPdiscussionwithhasband(1) by Q202Maindecideroncontraceptiveuse(2) | .929 | .268 | 12.051 | 1 | .001 | 2.532 | 1.499 | 4.279 |
|  | Q201FPdiscussionwithhasband(2) by Q202Maindecideroncontraceptiveuse(1) | 1.669 | 1.026 | 2.647 | 1 | .104 | 5.304 | .711 | 39.587 |
|  | Q201FPdiscussionwithhasband(2) by Q202Maindecideroncontraceptiveuse(2) | .937 | .301 | 9.678 | 1 | .002 | 2.551 | 1.414 | 4.603 |
|  | Constant | -1.669 | .227 | 53.875 | 1 | .000 | .189 |  |  |
| a. Variable(s) entered on step 1: Q201FPdiscussionwithhasband * Q202Maindecideroncontraceptiveuse . | | | | | | | | | |

| **Variables in the Equation(Income Vs Ownership of Radio/Television)** | | | | | | | | | |
| --- | --- | --- | --- | --- | --- | --- | --- | --- | --- |
|  | | B | S.E. | Wald | df | Sig. | Exp(B) | 95% C.I.for EXP(B) | |
|  |  |  |  |  |  |  |  | Lower | Upper |
| Step 1^a^ | Incomerecode * Q109Ownershipofredioortelevision |  |  | 1.940 | 3 | .585 |  |  |  |
|  | Incomerecode(1) by Q109Ownershipofredioortelevision(1) | .222 | .289 | .593 | 1 | .441 | 1.249 | .709 | 2.201 |
|  | Incomerecode(2) by Q109Ownershipofredioortelevision(1) | -.090 | .263 | .118 | 1 | .732 | .914 | .546 | 1.529 |
|  | Incomerecode(3) by Q109Ownershipofredioortelevision(1) | .244 | .272 | .801 | 1 | .371 | 1.276 | .748 | 2.175 |
|  | Constant | -1.038 | .173 | 35.767 | 1 | .000 | .354 |  |  |
| a. Variable(s) entered on step 1: Incomerecode * Q109Ownershipofredioortelevision . | | | | | | | | | |

| **Variables in the Equation (Women education Vs FP discussion)** | | | | | | | | | |
| --- | --- | --- | --- | --- | --- | --- | --- | --- | --- |
|  | | B | S.E. | Wald | df | Sig. | Exp(B) | 95% C.I.for EXP(B) | |
|  |  |  |  |  |  |  |  | Lower | Upper |
| Step 1^a^ | MergedEdu_W * Q201FPdiscussionwithhasband |  |  | 4.488 | 6 | .611 |  |  |  |
|  | MergedEdu_W(1) by Q201FPdiscussionwithhasband(1) | .235 | .322 | .532 | 1 | .466 | 1.265 | .672 | 2.380 |
|  | MergedEdu_W(1) by Q201FPdiscussionwithhasband(2) | .416 | .431 | .932 | 1 | .334 | 1.516 | .651 | 3.527 |
|  | MergedEdu_W(2) by Q201FPdiscussionwithhasband(1) | .498 | .326 | 2.341 | 1 | .126 | 1.646 | .869 | 3.115 |
|  | MergedEdu_W(2) by Q201FPdiscussionwithhasband(2) | .470 | .458 | 1.055 | 1 | .304 | 1.600 | .653 | 3.923 |
|  | MergedEdu_W(3) by Q201FPdiscussionwithhasband(1) | .504 | .351 | 2.064 | 1 | .151 | 1.655 | .832 | 3.292 |
|  | MergedEdu_W(3) by Q201FPdiscussionwithhasband(2) | .182 | .369 | .244 | 1 | .622 | 1.200 | .582 | 2.475 |
|  | Constant | -1.163 | .148 | 61.848 | 1 | .000 | .313 |  |  |
| a. Variable(s) entered on step 1: MergedEdu_W * Q201FPdiscussionwithhasband . | | | | | | | | | |

| **Variables in the Equation (Husband education Vs FP discussion)** | | | | | | | | | |
| --- | --- | --- | --- | --- | --- | --- | --- | --- | --- |
|  | | B | S.E. | Wald | df | Sig. | Exp(B) | 95% C.I.for EXP(B) | |
|  |  |  |  |  |  |  |  | Lower | Upper |
| Step 1^a^ | MergedEdu_H * Q201FPdiscussionwithhasband |  |  | 6.680 | 6 | .351 |  |  |  |
|  | MergedEdu_H(1) by Q201FPdiscussionwithhasband(1) | .527 | .315 | 2.798 | 1 | .094 | 1.693 | .914 | 3.138 |
|  | MergedEdu_H(1) by Q201FPdiscussionwithhasband(2) | .304 | .431 | .497 | 1 | .481 | 1.355 | .582 | 3.156 |
|  | MergedEdu_H(2) by Q201FPdiscussionwithhasband(1) | .528 | .306 | 2.987 | 1 | .084 | 1.696 | .932 | 3.089 |
|  | MergedEdu_H(2) by Q201FPdiscussionwithhasband(2) | -.193 | .528 | .133 | 1 | .715 | .825 | .293 | 2.322 |
|  | MergedEdu_H(3) by Q201FPdiscussionwithhasband(1) | .549 | .295 | 3.466 | 1 | .063 | 1.732 | .971 | 3.087 |
|  | MergedEdu_H(3) by Q201FPdiscussionwithhasband(2) | .174 | .392 | .198 | 1 | .656 | 1.191 | .552 | 2.566 |
|  | Constant | -1.242 | .177 | 49.096 | 1 | .000 | .289 |  |  |
| a. Variable(s) entered on step 1: MergedEdu_H * Q201FPdiscussionwithhasband . | | | | | | | | | |

| **Variables in the Equation (Women education Vs main decider on contraceptive use)** | | | | | | | | | |
| --- | --- | --- | --- | --- | --- | --- | --- | --- | --- |
|  | | B | S.E. | Wald | df | Sig. | Exp(B) | 95% C.I.for EXP(B) | |
|  |  |  |  |  |  |  |  | Lower | Upper |
| Step 1^a^ | MergedEdu_W * Q202Maindecideroncontraceptiveuse |  |  | 3.210 | 6 | .782 |  |  |  |
|  | MergedEdu_W(1) by Q202Maindecideroncontraceptiveuse(1) | -20.114 | 20096.485 | .000 | 1 | .999 | .000 | .000 | . |
|  | MergedEdu_W(1) by Q202Maindecideroncontraceptiveuse(2) | .113 | .282 | .162 | 1 | .688 | 1.120 | .644 | 1.947 |
|  | MergedEdu_W(2) by Q202Maindecideroncontraceptiveuse(1) | -20.114 | 28420.722 | .000 | 1 | .999 | .000 | .000 | . |
|  | MergedEdu_W(2) by Q202Maindecideroncontraceptiveuse(2) | .466 | .299 | 2.429 | 1 | .119 | 1.594 | .887 | 2.865 |
|  | MergedEdu_W(3) by Q202Maindecideroncontraceptiveuse(1) | -.010 | 1.163 | .000 | 1 | .993 | .990 | .101 | 9.679 |
|  | MergedEdu_W(3) by Q202Maindecideroncontraceptiveuse(2) | .337 | .280 | 1.449 | 1 | .229 | 1.401 | .809 | 2.427 |
|  | Constant | -1.089 | .140 | 60.306 | 1 | .000 | .337 |  |  |
| a. Variable(s) entered on step 1: MergedEdu_W * Q202Maindecideroncontraceptiveuse . | | | | | | | | | |

| **Variables in the Equation (Husband education Vs main decider on contraceptive use)** | | | | | | | | | |
| --- | --- | --- | --- | --- | --- | --- | --- | --- | --- |
|  | | B | S.E. | Wald | df | Sig. | Exp(B) | 95% C.I.for EXP(B) | |
|  |  |  |  |  |  |  |  | Lower | Upper |
| Step 1^a^ | MergedEdu_H * Q202Maindecideroncontraceptiveuse |  |  | 2.295 | 6 | .891 |  |  |  |
|  | MergedEdu_H(1) by Q202Maindecideroncontraceptiveuse(1) | .425 | .881 | .233 | 1 | .630 | 1.529 | .272 | 8.597 |
|  | MergedEdu_H(1) by Q202Maindecideroncontraceptiveuse(2) | .226 | .287 | .620 | 1 | .431 | 1.254 | .714 | 2.201 |
|  | MergedEdu_H(2) by Q202Maindecideroncontraceptiveuse(1) | 1.118 | 1.423 | .617 | 1 | .432 | 3.059 | .188 | 49.790 |
|  | MergedEdu_H(2) by Q202Maindecideroncontraceptiveuse(2) | .202 | .283 | .510 | 1 | .475 | 1.224 | .703 | 2.129 |
|  | MergedEdu_H(3) by Q202Maindecideroncontraceptiveuse(1) | -20.085 | 20096.485 | .000 | 1 | .999 | .000 | .000 | . |
|  | MergedEdu_H(3) by Q202Maindecideroncontraceptiveuse(2) | .327 | .262 | 1.554 | 1 | .213 | 1.387 | .829 | 2.318 |
|  | Constant | -1.118 | .161 | 48.043 | 1 | .000 | .327 |  |  |
| a. Variable(s) entered on step 1: MergedEdu_H * Q202Maindecideroncontraceptiveuse . | | | | | | | | | |

| **Variables in the Equation (Age Vs Birth intension)** | | | | | | | | | |
| --- | --- | --- | --- | --- | --- | --- | --- | --- | --- |
|  | | B | S.E. | Wald | df | Sig. | Exp(B) | 95% C.I.for EXP(B) | |
|  |  |  |  |  |  |  |  | Lower | Upper |
| Step 1^a^ | AGERecod * Q219Birthintension |  |  | 48.526 | 4 | .000 |  |  |  |
|  | AGERecod(1) by Q219Birthintension(1) | 1.965 | .313 | 39.383 | 1 | .000 | 7.132 | 3.861 | 13.173 |
|  | AGERecod(1) by Q219Birthintension(2) | 1.372 | .317 | 18.755 | 1 | .000 | 3.944 | 2.119 | 7.339 |
|  | AGERecod(2) by Q219Birthintension(1) | .964 | .617 | 2.446 | 1 | .118 | 2.623 | .783 | 8.781 |
|  | AGERecod(2) by Q219Birthintension(2) | 1.820 | .307 | 35.146 | 1 | .000 | 6.172 | 3.382 | 11.266 |
|  | Constant | -2.143 | .231 | 86.304 | 1 | .000 | .117 |  |  |
| a. Variable(s) entered on step 1: AGERecod * Q219Birthintension . | | | | | | | | | |

| **Variables in the Equation (Income Vs Women education)** | | | | | | | | | |
| --- | --- | --- | --- | --- | --- | --- | --- | --- | --- |
|  | | B | S.E. | Wald | df | Sig. | Exp(B) | 95% C.I.for EXP(B) | |
|  |  |  |  |  |  |  |  | Lower | Upper |
| Step 1^a^ | Incomerecode * MergedEdu_W |  |  | 5.278 | 9 | .809 |  |  |  |
|  | Incomerecode(1) by MergedEdu_W(1) | .469 | .422 | 1.237 | 1 | .266 | 1.599 | .699 | 3.656 |
|  | Incomerecode(1) by MergedEdu_W(2) | .084 | .608 | .019 | 1 | .890 | 1.087 | .330 | 3.578 |
|  | Incomerecode(1) by MergedEdu_W(3) | .440 | .642 | .471 | 1 | .493 | 1.553 | .441 | 5.466 |
|  | Incomerecode(2) by MergedEdu_W(1) | -.665 | .435 | 2.338 | 1 | .126 | .514 | .219 | 1.206 |
|  | Incomerecode(2) by MergedEdu_W(2) | .135 | .444 | .093 | 1 | .761 | 1.145 | .480 | 2.731 |
|  | Incomerecode(2) by MergedEdu_W(3) | -.099 | .458 | .046 | 1 | .830 | .906 | .369 | 2.223 |
|  | Incomerecode(3) by MergedEdu_W(1) | .189 | .617 | .094 | 1 | .759 | 1.208 | .361 | 4.047 |
|  | Incomerecode(3) by MergedEdu_W(2) | .253 | .428 | .349 | 1 | .555 | 1.288 | .557 | 2.978 |
|  | Incomerecode(3) by MergedEdu_W(3) | .182 | .340 | .286 | 1 | .593 | 1.199 | .616 | 2.333 |
|  | Constant | -1.000 | .139 | 51.906 | 1 | .000 | .368 |  |  |
| a. Variable(s) entered on step 1: Incomerecode * MergedEdu_W . | | | | | | | | | |

| **Variables in the Equation (Income Vs Husband education)** | | | | | | | | | |
| --- | --- | --- | --- | --- | --- | --- | --- | --- | --- |
|  | | B | S.E. | Wald | df | Sig. | Exp(B) | 95% C.I.for EXP(B) | |
|  |  |  |  |  |  |  |  | Lower | Upper |
| Step 1^a^ | Incomerecode * MergedEdu_H |  |  | 4.256 | 9 | .894 |  |  |  |
|  | Incomerecode(1) by MergedEdu_H(1) | .036 | .390 | .008 | 1 | .927 | 1.036 | .482 | 2.227 |
|  | Incomerecode(1) by MergedEdu_H(2) | .241 | .437 | .303 | 1 | .582 | 1.272 | .540 | 2.996 |
|  | Incomerecode(1) by MergedEdu_H(3) | .145 | .562 | .067 | 1 | .796 | 1.156 | .385 | 3.476 |
|  | Incomerecode(2) by MergedEdu_H(1) | -.607 | .476 | 1.623 | 1 | .203 | .545 | .214 | 1.386 |
|  | Incomerecode(2) by MergedEdu_H(2) | -.108 | .370 | .085 | 1 | .771 | .898 | .434 | 1.856 |
|  | Incomerecode(2) by MergedEdu_H(3) | -.424 | .405 | 1.098 | 1 | .295 | .654 | .296 | 1.447 |
|  | Incomerecode(3) by MergedEdu_H(1) | -.319 | .817 | .153 | 1 | .696 | .727 | .147 | 3.604 |
|  | Incomerecode(3) by MergedEdu_H(2) | .241 | .489 | .242 | 1 | .623 | 1.272 | .488 | 3.314 |
|  | Incomerecode(3) by MergedEdu_H(3) | .128 | .306 | .176 | 1 | .675 | 1.137 | .625 | 2.069 |
|  | Constant | -.934 | .156 | 35.669 | 1 | .000 | .393 |  |  |
| a. Variable(s) entered on step 1: Incomerecode * MergedEdu_H . | | | | | | | | | |

| **Variables in the Equation (Place of residence Vs myths heard)** | | | | | | | | | |
| --- | --- | --- | --- | --- | --- | --- | --- | --- | --- |
|  | | B | S.E. | Wald | df | Sig. | Exp(B) | 95% C.I.for EXP(B) | |
|  |  |  |  |  |  |  |  | Lower | Upper |
| Step 1^a^ | Q103PlaceofResidence(1) by Q208HaveyouhearedMyths(1) | .357 | .203 | 3.083 | 1 | .079 | 1.429 | .959 | 2.129 |
|  | Constant | -1.107 | .130 | 72.762 | 1 | .000 | .331 |  |  |
| a. Variable(s) entered on step 1: Q103PlaceofResidence * Q208HaveyouhearedMyths . | | | | | | | | | |

| **Variables in the Equation (Place of residence Vs Birth intension)** | | | | | | | | | |
| --- | --- | --- | --- | --- | --- | --- | --- | --- | --- |
|  | | B | S.E. | Wald | df | Sig. | Exp(B) | 95% C.I.for EXP(B) | |
|  |  |  |  |  |  |  |  | Lower | Upper |
| Step 1^a^ | Q103PlaceofResidence * Q219Birthintension |  |  | .029 | 2 | .986 |  |  |  |
|  | Q103PlaceofResidence(1) by Q219Birthintension(1) | -20.263 | 13397.657 | .000 | 1 | .999 | .000 | .000 | . |
|  | Q103PlaceofResidence(1) by Q219Birthintension(2) | -.090 | .531 | .029 | 1 | .866 | .914 | .323 | 2.588 |
|  | Constant | -.940 | .102 | 85.121 | 1 | .000 | .391 |  |  |
| a. Variable(s) entered on step 1: Q103PlaceofResidence * Q219Birthintension . | | | | | | | | | |

| **Variables in the Equation** | | | | | | | | | |
| --- | --- | --- | --- | --- | --- | --- | --- | --- | --- |
|  | | B | S.E. | Wald | df | Sig. | Exp(B) | 95% C.I.for EXP(B) | |
|  |  |  |  |  |  |  |  | Lower | Upper |
| Step 1^a^ | AGERecod |  |  | .052 | 2 | .974 |  |  |  |
|  | AGERecod(1) | .229 | 1.004 | .052 | 1 | .819 | 1.258 | .176 | 8.994 |
|  | AGERecod(2) | .187 | 1.139 | .027 | 1 | .870 | 1.205 | .129 | 11.245 |
|  | Q103PlaceofResidence(1) | .920 | .594 | 2.399 | 1 | .121 | 2.509 | .783 | 8.033 |
|  | Q105Occupation |  |  | 2.859 | 4 | .582 |  |  |  |
|  | Q105Occupation(1) | .481 | .385 | 1.562 | 1 | .211 | 1.617 | .761 | 3.437 |
|  | Q105Occupation(2) | -.002 | .530 | .000 | 1 | .996 | .998 | .353 | 2.820 |
|  | Q105Occupation(3) | .393 | .394 | .995 | 1 | .319 | 1.482 | .684 | 3.208 |
|  | Q105Occupation(4) | .713 | .745 | .917 | 1 | .338 | 2.041 | .474 | 8.787 |
|  | ReligionRecoded |  |  | .361 | 2 | .835 |  |  |  |
|  | ReligionRecoded(1) | .126 | .468 | .073 | 1 | .788 | 1.134 | .453 | 2.840 |
|  | ReligionRecoded(2) | .342 | .600 | .325 | 1 | .569 | 1.407 | .434 | 4.558 |
|  | MergedEdu_W |  |  | .440 | 3 | .932 |  |  |  |
|  | MergedEdu_W(1) | .220 | .375 | .342 | 1 | .559 | 1.246 | .597 | 2.599 |
|  | MergedEdu_W(2) | .200 | .437 | .210 | 1 | .647 | 1.222 | .519 | 2.879 |
|  | MergedEdu_W(3) | .248 | .569 | .190 | 1 | .663 | 1.281 | .420 | 3.911 |
|  | MergedEdu_H |  |  | .421 | 3 | .936 |  |  |  |
|  | MergedEdu_H(1) | .139 | .387 | .129 | 1 | .719 | 1.149 | .538 | 2.456 |
|  | MergedEdu_H(2) | .082 | .426 | .037 | 1 | .847 | 1.086 | .471 | 2.505 |
|  | MergedEdu_H(3) | -.125 | .443 | .080 | 1 | .777 | .882 | .370 | 2.102 |
|  | Incomerecode |  |  | 1.163 | 3 | .762 |  |  |  |
|  | Incomerecode(1) | .181 | .385 | .222 | 1 | .638 | 1.199 | .564 | 2.547 |
|  | Incomerecode(2) | .368 | .388 | .901 | 1 | .342 | 1.445 | .676 | 3.090 |
|  | Incomerecode(3) | .065 | .467 | .019 | 1 | .890 | 1.067 | .427 | 2.666 |
|  | Q110LACadvertismentheard(1) | -1.252 | .669 | 3.499 | 1 | .061 | .286 | .077 | 1.062 |
|  | ARTstatus(1) | 1.059 | .344 | 9.453 | 1 | .002 | 2.882 | 1.468 | 5.660 |
|  | Q201FPdiscussionwithhasband |  |  | 8.947 | 2 | .011 |  |  |  |
|  | Q201FPdiscussionwithhasband(1) | 2.085 | .698 | 8.938 | 1 | .003 | 8.048 | 2.051 | 31.584 |
|  | Q201FPdiscussionwithhasband(2) | 1.582 | 1.473 | 1.153 | 1 | .283 | 4.864 | .271 | 87.273 |
|  | Q202Maindecideroncontraceptiveuse |  |  | .231 | 2 | .891 |  |  |  |
|  | Q202Maindecideroncontraceptiveuse(1) | .523 | 1.318 | .157 | 1 | .692 | 1.687 | .127 | 22.346 |
|  | Q202Maindecideroncontraceptiveuse(2) | .420 | 1.293 | .106 | 1 | .745 | 1.522 | .121 | 19.169 |
|  | Q207LACMspastexperience(1) | 2.193 | .295 | 55.252 | 1 | .000 | 8.959 | 5.026 | 15.973 |
|  | Q208HaveyouhearedMyths(1) | .744 | .277 | 7.200 | 1 | .007 | 2.104 | 1.222 | 3.621 |
|  | AlivechildrenRe_Cat(1) | .430 | .361 | 1.419 | 1 | .234 | 1.538 | .758 | 3.120 |
|  | Q219Birthintension |  |  | 3.925 | 2 | .140 |  |  |  |
|  | Q219Birthintension(1) | 2.234 | 1.128 | 3.918 | 1 | .048 | 9.333 | 1.022 | 85.232 |
|  | Q219Birthintension(2) | 1.620 | 1.362 | 1.416 | 1 | .234 | 5.055 | .350 | 72.909 |
|  | Q109Ownershipofredioortelevision(1) | 1.202 | .590 | 4.152 | 1 | .042 | 3.327 | 1.047 | 10.570 |
|  | Q201FPdiscussionwithhasband * Q202Maindecideroncontraceptiveuse |  |  | 1.031 | 4 | .905 |  |  |  |
|  | Q201FPdiscussionwithhasband(1) by Q202Maindecideroncontraceptiveuse(1) | -1.664 | 1.830 | .826 | 1 | .363 | .189 | .005 | 6.842 |
|  | Q201FPdiscussionwithhasband(1) by Q202Maindecideroncontraceptiveuse(2) | -.265 | 1.372 | .037 | 1 | .847 | .767 | .052 | 11.288 |
|  | Q201FPdiscussionwithhasband(2) by Q202Maindecideroncontraceptiveuse(1) | .032 | 2.247 | .000 | 1 | .988 | 1.033 | .013 | 84.423 |
|  | Q201FPdiscussionwithhasband(2) by Q202Maindecideroncontraceptiveuse(2) | .380 | 1.871 | .041 | 1 | .839 | 1.463 | .037 | 57.296 |
|  | AGERecod * Q219Birthintension |  |  | 2.065 | 4 | .724 |  |  |  |
|  | AGERecod(1) by Q219Birthintension(1) | -.284 | 1.221 | .054 | 1 | .816 | .753 | .069 | 8.241 |
|  | AGERecod(1) by Q219Birthintension(2) | .123 | 1.432 | .007 | 1 | .931 | 1.131 | .068 | 18.740 |
|  | AGERecod(2) by Q219Birthintension(1) | -1.049 | 1.471 | .508 | 1 | .476 | .350 | .020 | 6.265 |
|  | AGERecod(2) by Q219Birthintension(2) | .437 | 1.517 | .083 | 1 | .773 | 1.549 | .079 | 30.289 |
|  | Constant | -7.542 | 1.346 | 31.389 | 1 | .000 | .001 |  |  |
| a. Variable(s) entered on step 1: AGERecod, Q103PlaceofResidence, Q105Occupation, ReligionRecoded, MergedEdu_W, MergedEdu_H, Incomerecode, Q110LACadvertismentheard, ARTstatus, Q201FPdiscussionwithhasband, Q202Maindecideroncontraceptiveuse, Q207LACMspastexperience, Q208HaveyouhearedMyths, AlivechildrenRe_Cat, Q219Birthintension, Q109Ownershipofredioortelevision, Q201FPdiscussionwithhasband * Q202Maindecideroncontraceptiveuse , AGERecod * Q219Birthintension . | | | | | | | | | |
