# Supplementary material for: Factors affecting long acting and permanent contraceptive methods utilization among HIV positive married women attending care at ART clinics in Northwest Ethiopia
Source: Arch Public Health. 2018 Jul 16;76:47. doi: 10.1186/s13690-018-0294-0 (PMC6047118; doi:10.1186/s13690-018-0294-0)
Supplement: Supplementary file 3 — Hosmer and Lemeshow Test and the multivariable analysis output. (DOCX 27 kb) [file 13690_2018_294_MOESM3_ESM.docx]

**Multivariable analysis output**

| **Hosmer and Lemeshow Test** | | | |
| --- | --- | --- | --- |
| Step | Chi-square | df | Sig. |
| 1 | 2.371 | 8 | .967 |
| 12 | 4.300 | 8 | .829 |

| **Variables in the Equation** | | | | | | | | | |
| --- | --- | --- | --- | --- | --- | --- | --- | --- | --- |
|  | | B | S.E. | Wald | df | Sig. | Exp(B) | 95% C.I.for EXP(B) | |
|  |  |  |  |  |  |  |  | Lower | Upper |
| Step 1^a^ | AGERecod |  |  | .062 | 2 | .970 |  |  |  |
|  | AGERecod(1) | .247 | 1.008 | .060 | 1 | .807 | 1.280 | .178 | 9.220 |
|  | AGERecod(2) | .244 | 1.141 | .046 | 1 | .831 | 1.276 | .136 | 11.947 |
|  | Q103PlaceofResidence(1) | .805 | .574 | 1.965 | 1 | .161 | 2.237 | .726 | 6.894 |
|  | Q105Occupation |  |  | 3.946 | 4 | .413 |  |  |  |
|  | Q105Occupation(1) | .564 | .380 | 2.207 | 1 | .137 | 1.758 | .835 | 3.700 |
|  | Q105Occupation(2) | .057 | .528 | .012 | 1 | .914 | 1.058 | .376 | 2.977 |
|  | Q105Occupation(3) | .432 | .390 | 1.227 | 1 | .268 | 1.540 | .717 | 3.305 |
|  | Q105Occupation(4) | .907 | .737 | 1.513 | 1 | .219 | 2.477 | .584 | 10.508 |
|  | ReligionRecoded |  |  | .330 | 2 | .848 |  |  |  |
|  | ReligionRecoded(1) | .116 | .469 | .061 | 1 | .804 | 1.123 | .448 | 2.814 |
|  | ReligionRecoded(2) | .327 | .598 | .300 | 1 | .584 | 1.387 | .430 | 4.476 |
|  | MergedEdu_W |  |  | .389 | 3 | .942 |  |  |  |
|  | MergedEdu_W(1) | .210 | .369 | .323 | 1 | .570 | 1.234 | .598 | 2.545 |
|  | MergedEdu_W(2) | .187 | .433 | .186 | 1 | .666 | 1.206 | .516 | 2.819 |
|  | MergedEdu_W(3) | .202 | .565 | .128 | 1 | .720 | 1.224 | .405 | 3.704 |
|  | MergedEdu_H |  |  | .384 | 3 | .943 |  |  |  |
|  | MergedEdu_H(1) | .086 | .381 | .050 | 1 | .822 | 1.089 | .516 | 2.300 |
|  | MergedEdu_H(2) | .045 | .419 | .012 | 1 | .914 | 1.046 | .461 | 2.377 |
|  | MergedEdu_H(3) | -.169 | .435 | .150 | 1 | .698 | .845 | .360 | 1.982 |
|  | Incomerecode |  |  | 1.026 | 3 | .795 |  |  |  |
|  | Incomerecode(1) | -.014 | .462 | .001 | 1 | .975 | .986 | .399 | 2.438 |
|  | Incomerecode(2) | .161 | .447 | .130 | 1 | .719 | 1.175 | .490 | 2.818 |
|  | Incomerecode(3) | .306 | .396 | .598 | 1 | .439 | 1.358 | .625 | 2.948 |
|  | Q110LACadvertismentheard(1) | -.421 | .518 | .660 | 1 | .417 | .656 | .238 | 1.812 |
|  | ARTstatus(1) | 1.079 | .341 | 9.984 | 1 | .002 | 2.942 | 1.506 | 5.745 |
|  | Q201FPdiscussionwithhasband |  |  | 1.455 | 2 | .483 |  |  |  |
|  | Q201FPdiscussionwithhasband(1) | 1.281 | 1.137 | 1.269 | 1 | .260 | 3.599 | .388 | 33.387 |
|  | Q201FPdiscussionwithhasband(2) | 1.375 | 1.144 | 1.445 | 1 | .229 | 3.956 | .420 | 37.260 |
|  | Q202Maindecideroncontraceptiveuse |  |  | .439 | 2 | .803 |  |  |  |
|  | Q202Maindecideroncontraceptiveuse(1) | -.796 | 1.227 | .421 | 1 | .516 | .451 | .041 | 4.992 |
|  | Q202Maindecideroncontraceptiveuse(2) | -.489 | 1.657 | .087 | 1 | .768 | .613 | .024 | 15.761 |
|  | Q207LACMspastexperience(1) | 2.149 | .292 | 54.280 | 1 | .000 | 8.580 | 4.844 | 15.200 |
|  | Q208HaveyouhearedMyths(1) | .736 | .276 | 7.117 | 1 | .008 | 2.087 | 1.216 | 3.582 |
|  | AlivechildrenRe_Cat(1) | .410 | .359 | 1.307 | 1 | .253 | 1.507 | .746 | 3.046 |
|  | Q219Birthintention |  |  | 3.969 | 2 | .137 |  |  |  |
|  | Q219Birthintention (1) | 2.250 | 1.131 | 3.956 | 1 | .047 | 9.490 | 1.033 | 87.155 |
|  | Q219Birthintention (2) | 1.674 | 1.367 | 1.500 | 1 | .221 | 5.334 | .366 | 77.725 |
|  | Q201FPdiscussionwithhasband * Q202Maindecideroncontraceptiveuse |  |  | .952 | 4 | .917 |  |  |  |
|  | Q201FPdiscussionwithhasband(1) by Q202Maindecideroncontraceptiveuse(1) | .646 | 1.310 | .244 | 1 | .622 | 1.908 | .147 | 24.849 |
|  | Q201FPdiscussionwithhasband(1) by Q202Maindecideroncontraceptiveuse(2) | -.769 | 2.060 | .139 | 1 | .709 | .464 | .008 | 26.269 |
|  | Q201FPdiscussionwithhasband(2) by Q202Maindecideroncontraceptiveuse(1) | -.012 | 1.814 | .000 | 1 | .995 | .988 | .028 | 34.549 |
|  | Q201FPdiscussionwithhasband(2) by Q202Maindecideroncontraceptiveuse(2) | .245 | 2.073 | .014 | 1 | .906 | 1.277 | .022 | 74.329 |
|  | AGERecod * Q219Birthintention |  |  | 1.991 | 4 | .737 |  |  |  |
|  | AGERecod(1) by Q219Birthintention (1) | -.243 | 1.222 | .039 | 1 | .843 | .785 | .072 | 8.607 |
|  | AGERecod(1) by Q219Birthintention (2) | .147 | 1.435 | .011 | 1 | .918 | 1.159 | .070 | 19.293 |
|  | AGERecod(2) by Q219Birthintention (1) | -1.061 | 1.472 | .519 | 1 | .471 | .346 | .019 | 6.198 |
|  | AGERecod(2) by Q219Birthintention (2) | .395 | 1.520 | .068 | 1 | .795 | 1.485 | .075 | 29.205 |
|  | Constant | -6.397 | 1.679 | 14.517 | 1 | .000 | .002 |  |  |
| Step 12^a^ | Q103PlaceofResidence(1) | .960 | .510 | 3.538 | 1 | .060 | 2.611 | .960 | 7.100 |
|  | ARTstatus(1) | .975 | .310 | 9.900 | 1 | .002 | 2.650 | 1.444 | 4.863 |
|  | Q201FPdiscussionwithhasband |  |  | 16.002 | 2 | .000 |  |  |  |
|  | Q201FPdiscussionwithhasband(1) | 1.796 | .466 | 14.884 | 1 | .000 | 6.027 | 2.420 | 15.009 |
|  | Q201FPdiscussionwithhasband(2) | 1.888 | .496 | 14.486 | 1 | .000 | 6.608 | 2.499 | 17.472 |
|  | Q207LACMspastexperience(1) | 2.204 | .266 | 68.621 | 1 | .000 | 9.057 | 5.377 | 15.256 |
|  | Q208HaveyouhearedMyths(1) | .728 | .261 | 7.795 | 1 | .005 | 2.070 | 1.242 | 3.449 |
|  | Q219Birthintention |  |  | 35.127 | 2 | .000 |  |  |  |
|  | Q219Birthintention (1) | 1.938 | .373 | 27.025 | 1 | .000 | 6.945 | 3.345 | 14.421 |
|  | Q219Birthintention (2) | 2.029 | .358 | 32.100 | 1 | .000 | 7.604 | 3.769 | 15.341 |
|  | Constant | -6.379 | .744 | 73.465 | 1 | .000 | .002 |  |  |
| a. Variable(s) entered on step 1: AGERecod, Q103PlaceofResidence, Q105Occupation, ReligionRecoded, MergedEdu_W, MergedEdu_H, Incomerecode, Q110LACadvertismentheard, ARTstatus, Q201FPdiscussionwithhasband, Q202Maindecideroncontraceptiveuse, Q207LACMspastexperience, Q208HaveyouhearedMyths, AlivechildrenRe_Cat, Q219Birthintention , Q201FPdiscussionwithhasband * Q202Maindecideroncontraceptiveuse , AGERecod * Q219Birthintention . | | | | | | | | | |
